# Population seroprevalence of antibody to influenza A(H7N9) virus, Guangzhou, China

**DOI:** 10.1186/s12879-016-1983-3

**Published:** 2016-11-04

**Authors:** Yong Ping Lin, Zi Feng Yang, Ying Liang, Zheng Tu Li, Helen S. Bond, Huiying Chua, Ya Sha Luo, Yuan Chen, Ting Ting Chen, Wen Da Guan, Jimmy Chun Cheong Lai, Yu Lam Siu, Si Hua Pan, J. S. Malik Peiris, Benjamin J. Cowling, Chris Ka PunMok

**Affiliations:** 1 Department of Laboratory Medicine, The First Affiliated Hospital of Guangzhou Medical University, Guangdong, China; 2 Research Centre of Translational Medicine, The First Affiliated Hospital of Guangzhou Medical University, Guangdong, China; 3 State Key Laboratory of Respiratory Disease, National Clinical Research Center for Respiratory Disease, First Affiliated Hospital of Guangzhou Medical University, Guangdong, China; 4 WHO Collaborating Centre for Infectious Disease Epidemiology and Control, School of Public Health, Li Ka Shing Faculty of Medicine, The University of Hong Kong, Hong Kong Special Administrative Region, China; 5 Department of Laboratory Medicine, The Second Affiliated Hospital of Guangzhou University of Chinese Medicine, Guangdong, China; 6 Department of Pathology, Li Ka Shing Faculty of Medicine, The University of Hong Kong, Hong Kong Special Administrative Region, China; 7 HKU-Pasteur Research Pole, School of Public Health, Li Ka Shing Faculty of Medicine, The University of Hong Kong, Hong Kong Special Administrative Region, China; 8 Centre of Influenza Research, School of Public Health, Li Ka Shing Faculty of Medicine, The University of Hong Kong, Hong Kong Special Administrative Region, China; 9 School of Public Health, Li Ka Shing Faculty of Medicine, The University of Hong Kong, 21 Sassoon Road, Pokfulam, Hong Kong, China

**Keywords:** Avian influenza A(H7N9), Public health, Serology, Severity

## Abstract

**Background:**

Since the identification in early 2013 of severe disease caused by influenza A(H7N9) virus infection, there have been few attempts to characterize the full severity profile of human infections. Our objective was to estimate the number and severity of H7N9 infections in Guangzhou, using a serological study.

**Methods:**

We collected residual sera from patients of all ages admitted to a hospital in the city of Guangzhou in southern China in 2013 and 2014. We screened the sera using a haemagglutination inhibition assay against a pseudovirus containing the H7 and N9 of A/Anhui/1/2013(H7N9), and samples with a screening titer ≥10 were further tested by standard hemagglutination-inhibition and virus neutralization assays for influenza A(H7N9). We used a statistical model to interpret the information on antibody titers in the residual sera, assuming that the residual sera provided a representative picture of A(H7N9) infections in the general population, accounting for potential cross-reactions.

**Results:**

We collected a total of 5360 residual sera from December 2013 to April 2014 and from October 2014 to December 2014, and found two specimens that tested positive for H7N9 antibody at haemagglutination inhibition titer ≥40 and a neutralization titer ≥40. Based on this, we estimated that 64,000 (95 % credibility interval: 7300, 190,000) human infections with influenza A(H7N9) virus occurred in Guangzhou in early 2014, with an infection-fatality risk of 3.6 deaths (95 % credibility interval: 0.47, 15) per 10,000 infections.

**Conclusions:**

Our study suggested that the number of influenza A(H7N9) virus infections in Guangzhou substantially exceeded the number of laboratory-confirmed cases there, albeit with considerable imprecision. Our study was limited by the small number of positive specimens identified, and larger serologic studies would be valuable. Our analytic framework would be useful if larger serologic studies are done.

**Electronic supplementary material:**

The online version of this article (doi:10.1186/s12879-016-1983-3) contains supplementary material, which is available to authorized users.

## Background

A novel avian influenza A(H7N9) virus emerged in early 2013 [1, 2] and caused cases of human infections in China in spring 2013, and subsequent winter and spring periods. The first epidemic wave was concentrated in eastern China, but Guangdong province in southern China was one of the most heavily affected areas in the second epidemic wave of H7N9 between December 2013 and April 2014 [3]. In total, 124 severe cases were reported in the first wave and 273 severe cases in the second wave, across China [3]. Guangzhou, a city with population 12.8 million in 2013, is the provincial capital of Guangdong province. The objective of this study was to estimate the number of human infections with H7N9 virus in Guangzhou, and infer the severity of human infections on a per-infection basis.

## Methods

### Recruitment of participants

We collected residual sera in the First Affiliated Hospital of Guangzhou Medical University from patients in non-respiratory diseases wards with no signs of influenza-like illness at presentation or during hospitalization, and patients from the routine body check center. Sera were collected in two phases: 2674 sera were collected from December 2013 to April 2014, and 2686 sera were collected from October 2014 to December 2014.

### Laboratory methods

Residual sera were first tested in parallel in each phase, using haemagglutination inhibition (HI) assay against a pseudovirus containing the H7 and N9 of A/Anhui/1/2013(H7N9) using methods previously described [4]. Serum from a laboratory-confirmed H7N9 case was used as a positive control. Then, 306 positive samples with HI titer ≥10 on the pseudovirus assay were further tested with HI and neutralization assays using live A/Anhui/1/2013(H7N9) viruses in a BSL-3 laboratory according to the guidelines from WHO (HI: http://www.who.int/influenza/gisrs_laboratory/cnic_serological_diagnosis_hai_a_h7n9_20131220.pdf; MN: http://www.who.int/influenza/gisrs_laboratory/cnic_serological_diagnosis_microneutralization_a_h7n9.pdf) using 1 % horse red blood cells. We defined a positive serum sample as a sample with a titer ≥40 by HI and MN.

### Statistical analysis

We analyzed the serological data to infer the cumulative incidence of influenza A(H7N9) infections, in a Bayesian framework. In the Bayesian framework, information brought by new data, in the form of the likelihood function, is combined with prior information that is specified in a prior distribution to obtain a posterior distribution that represents updated knowledge.

We stratified our analyses by age group (0–14, 15–24, 25–54, 55–64 and 65+ years of age) to allow extrapolation of seroprevalence to the underlying population. We described the weekly probability of a serum being tested positive (i.e. infected) for each age group as a parameter that depended on the baseline seroprevalence plus the seroprevalence due to recent infections. We assumed that all infected individuals had a rise in titer to ≥40 after a lag of 2 weeks [5], and that titers following infection waned after 6 months because that occurs following other infections including human influenza virus infections [6, 7]. We did not have information on the timing of infections in the general population, but we did have information on the timing of laboratory-confirmed cases which were generally severe and required hospitalization. We assumed that the risk of severe disease remained a constant fraction of all infections over the course of the epidemic, so that the timing of severe cases was indicative of the timing of infections in the general population (Additional file 1). Based on these assumptions, we described the seroprevalence due to recent infections as a product of the cumulative incidence of recent H7N9 infections and expected scaled seroprevalence based on the delay between possible infection dates and collection of sera. Yang et al. [5] reported that before the H7N9 epidemic, 9/1129 specimens collected in April-May 2013 from the general population in Zhejiang province were positive for H7N9 at a HI titer of ≥40. We used this information to construct informative priors for the baseline seroprevalence in each age group at a titer ≥40 based on the population structure in Guangzhou (Additional file 1). We used a Jeffrey’s prior, i.e. a beta(0.5, 0.5) distribution, as a non-informative prior distribution for the cumulative incidence of infection in each age group. We also conducted a sensitivity analysis using a flat beta(1, 1) prior instead for the cumulative incidence of infection.

We fitted the model using Markov Chain Monte Carlo (MCMC) methods with a warm-up period of 30,000 iterations followed by a further 30,000 iterations and five chains. Posterior estimates of the cumulative incidence of infection in each age group were then combined using population weights to provide a single estimate of the overall age-standardized cumulative incidence of H7N9 infection in Guangzhou. Given this estimate, we further estimated the total number of infections in the population.

In a previously published study [8], 16 severe cases of H7N9 were identified through routine surveillance of pneumonia of unknown etiology from January 2014 to March 2014. No severe cases of H7N9 were reported in Guangzhou from October 2014 to December 2014. In addition to national surveillance, we conducted a surveillance study in our hospital from March 2014 to August 2014 and found no positive cases of H7N9 in 324 hospitalized patients and 1012 influenza-like illness (ILI) cases. We used this information to estimate the risk of severe case and death following an H7N9 infection. We used trace plots and the potential scale reduction statistic [9] to confirm that MCMC chains converged and were well-mixed. All statistical analyses were conducted using R version 3.1.2 (R Foundation for Statistical Computing, Vienna, Austria).

## Results

**Fig. 1:**
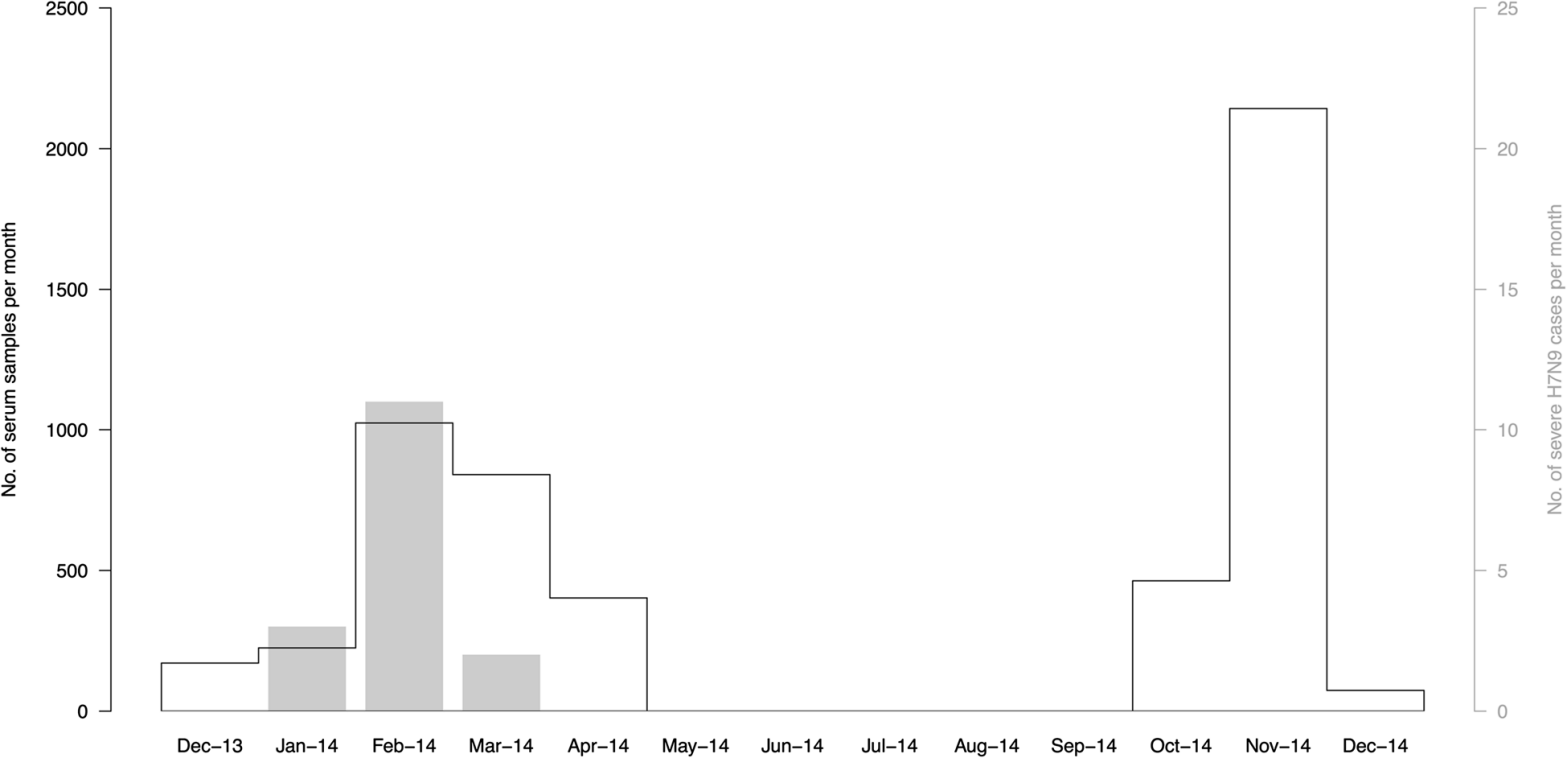
Timing of collection of residual sera from 5340 patients in Guangzhou in two phases (black lines), and the detection of severe cases of infection with influenza A(H7N9) virus in Guangzhou (gray bars) described in a separate study [8]

The age and sex distribution of patients from whom we collected residual sera is shown in Table 1 and the timing of sera collection in comparison to the timing of detection of severe cases in Guangzhou are shown in Fig. 1. Of 306 positive samples with HI titer ≥10 on the pseudovirus assay, two specimens further tested positive for H7N9 antibody at an HI titer of ≥40 and a neutralization titer ≥40: a specimen collected on 14 February 2014 from a 38y woman with HI titer of 1:80 on pseudovirus assay was positive at an HI titer of 80 and a neutralization titer of 40, and a specimen collected on 4 April 2014 from a 68y man with HI titer of 1:80 on pseudovirus assay was positive at an HI titer of 80 and a neutralization titer of 80. In addition, a specimen collected on 7 March 2014 from an 83y woman had HI titer of 1:40 on pseudovirus assay and was tested positive at an HI titer of 40 but had a neutralization titer ≤10. There was no report of recent acute respiratory illnesses in the 3 individuals with titers ≥40.

**Table 1:** Age and sex distribution of residual sera collected from Guangzhou

Characteristic	Phase 1 (December 2013 through April 2014) (*n* = 2660)	Phase 2 (October 2014 through December 2014) (*n* = 2680)
Age group, years		
0–14	185 (7.0 %)	140 (5.2 %)
15–24	415 (15.6 %)	445 (16.6 %)
25–54	1137 (42.7 %)	1166 (43.5 %)
55–64	340 (12.8 %)	397 (14.8 %)
≥ 65	583 (21.9 %)	532 (19.9 %)
Sex		
Male	1336 (50.2 %)	1240 (46.3 %)
Female	1324 (49.8 %)	1440 (53.7 %)

**Fig. 2:**
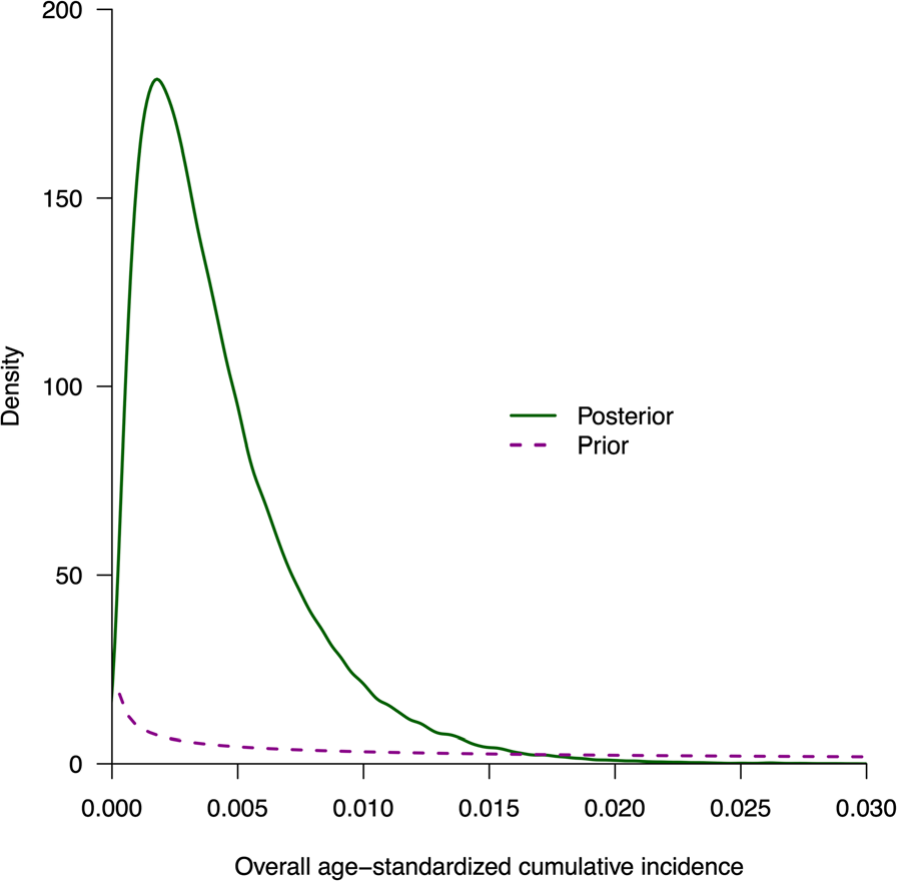
The prior and posterior distributions of the overall age-standardized cumulative incidence of H7N9 infections, assuming that antibody titers wane exactly 6 months (26 weeks) after infection. The most credible estimate of the overall age-standardized cumulative incidence of H7N9 infections between December 2013 and April 2014 was 0.43 % (95 % credibility interval: 0.05, 1.32 %)

We used MCMC to fit our model with a total of 60,000 iterations and 5 chains, and in diagnostic checking we confirmed that the MCMC runs were well-mixed and converged. Based on the two specimens that tested positive for H7N9 antibody at an HI titer of ≥40 and a neutralization titer ≥40, the posterior distribution (Fig. 2) indicates that the most credible estimate of the overall age-standardized cumulative incidence of H7N9 infections between December 2013 and April 2014 was 0.50 % (95 % credibility interval, CrI: 0.06, 1.51 %), corresponding to 64,000 (95 % CrI: 7300, 190,000) infections in Guangzhou (Table 2). Using this as the denominator, and the 16 severe cases and 11 deaths as the numerators respectively [8], we estimated that the risk of a severe illness following infection was 5.2 (95 % CrI: 0.72, 23) per 10,000 infections while the risk of death following infection was 3.6 (95 % CrI: 0.47, 15) per 10,000 infections. Alternative assumptions about the waning in antibody titers after infection led to broadly similar estimates (Table 2). Scenario 3 gave a somewhat lower estimate of the cumulative incidence of infection and higher estimates of risk of severe case and death following an infection.

**Table 2:** Results of main analyses and sensitivity analyses

	Main analysis				Sensitivity analysis	
	Alternative models including 2 specimens with HI and neutralization titers ≥40				Main model with alternative prior for cumulative incidence	Main model including three specimens with HI titers ≥40
Model	1	2	3	4	1	1
Estimated overall cumulative incidence, *θ* (%)	0.43 (0.05, 1.32)	0.52 (0.06, 1.59)	0.17 (0.02, 0.50)	0.41 (0.06, 1.22)	0.40 (0.06, 1.17)	0.43 (0.05, 1.32)
Estimated total number of infections	55,385 (6503, 169,010)	66,441 (7572, 204,113)	21,638 (2910, 64,431)	52,507 (7387, 156,391)	51,644 (8091, 149,142)	55,481 (6576, 168,665)
ISR (per 10,000 infections)	5.90 (0.84, 25.11)	5.02 (0.69, 21.68)	14.17 (2.17, 56.43)	5.66 (0.90, 22.40)	5.82 (1.00, 21.71)	5.89 (0.83, 24.83)
IFR (per 10,000 infections)	4.07 (0.54, 17.41)	3.45 (0.44, 15.13)	9.75 (1.41, 39.48)	3.89 (0.58, 15.69)	4.11 (0.68, 15.68)	4.06 (0.54, 17.49)

The main analysis was repeated to compare four alternative assumptions of expected scaled seroprevalence, *x* ' _*i*_. Sensitivity analyses aimed at comparing 1. main models using Jeffrey’s prior distribution (beta(0.5, 0.5)) versus flat beta prior distribution (i.e. beta(1, 1)) for cumulative incidence, *θ*, and 2. main models which considered two sera versus three sera that tested positive for H7N9 at a HI titer of ≥40. Note: All results are expressed in the most credible estimate (95 % credibility interval), *ISR* infection-severity risk, *IFR* infection-fatality risk

In a sensitivity analysis using a beta(1, 1) prior for the cumulative incidence of infection, we estimated that there were 59,000 (95 % CrI: 9300, 170,000) infections, and the risk of death following infection was 3.6 (95 % CrI: 0.60, 14) per 10,000 infections. In another sensitivity analysis including the three samples with HI titers of ≥40 as positives, we estimated that there were 64,000 (95 % CrI: 7400, 190,000) infections, and the risk of death following infection was 3.5 (95 % CrI: 0.47, 15) per 10,000 infections.

## Discussion

In this study we estimated that 64,000 (95 % credibility interval: 7300, 190,000) humans were infected with influenza A(H7N9) virus in Guangzhou between January 2014 and April 2014, which far exceeds the 20 laboratory-confirmed infections [8]. It was previously estimated that the number of cases of symptomatic H7N9 virus infections could be around 500–1000 times the number of laboratory-confirmed cases [3, 10], and this is consistent with our estimates of the numbers of infections albeit with considerable uncertainty. We are the first to estimate the infection fatality risk for H7N9, and our estimate of 3.6 deaths (95 % CrI: 0.47, 15) per 10,000 infections places the severity of H7N9 higher than that of human influenza viruses for which the fatality risk is around 1 per 10,000 infections but varies considerably by age [11]. The severity of H7N9 virus infections appears to increase with age [3, 10], but we did not have sufficient sample size to make robust age-specific estimates of incidence or severity. We would anticipate higher incidence of H7N9 infections in adult women and older adult men, based on patterns in exposure frequency in live poultry markets [12].

Our study is limited by the small number of positive samples. We used a statistical model to extrapolate from the small number of positives to a population estimate of the cumulative incidence of infection, which had very considerable uncertainty. Because we did not collect detailed patient information such as exposures when obtaining the sera specimens, we were unable to explore risk factors for infection. Larger serological studies in affected areas would be worthwhile, and the methods we describe could provide a framework for analysis of such studies. However, since larger serologic studies (i.e. with tens of thousands or even hundreds of thousands of samples) have not been reported, and to the best of our knowledge have not been done, our data may provide the only available population-based estimates of the cumulative incidence of H7N9 infection.

Several limitations concerning study design bear mention. Firstly, as most of our samples from the first sample collection were obtained before or during the peak incidence of H7N9 cases in Guangzhou and second sample collection was initiated in October 2014, the population exposure to influenza A H7N9 may not be optimally captured. In hindsight, it would have been more ideal to arrange collection of sera in April to June 2014, the period when maximal serological responses to H7N9 exposure might have been expected. We accounted for the overlapping of epidemic and sera collection in our analysis. Secondly, instead of analyzing serial cross-sectional residual sera from hospitals, collecting paired sera before and after epidemics would allow better identification of recent infections based on rises in antibody titers. However such a study would be much more complex and challenging than the one we conducted. Thirdly, collection of sera directly from the general community, rather than through hospitals, would be preferable if feasible, to minimize potential selection biases. Fourthly, antibody titers following laboratory-confirmed H7N9 virus infection can be low, and some infections might have been missed. One study that followed up severe cases reported that 65.8 % of non-fatal cases had detectable H7N9 antibody, 14 days after illness onset [5]. Finally, while a titer ≥40 is not conclusive evidence of infection because of the potential for a small number of people to have cross-reactive antibody following historical infections with other viruses [13], we accounted for this baseline seroprevalence in our model.

Our estimate of the total number of infections, including mild or asymptomatic infections, is consistent with the information available from ILI surveillance in Guangzhou. In national surveillance in the first 10 weeks of 2014 there were 58,291 ILI cases in sentinel clinics, of whom 1090 cases were tested, and three were positive for H7N9 [8]. While it is not possible to make a population-based estimate of the number of mild cases based only on this information, it is clear that the occurrence of ILI due to H7N9 infection in early 2014 was around two orders of magnitude lower than the expected occurrence of ILI due to seasonal human influenza activity in a typical epidemic when perhaps 30 % of ILI cases could test positive for human influenza. At a population level, 10 to 20 % of persons might be infected with human influenza viruses during an epidemic, and our estimate is consistent with the incidence of H7N9 infections being two orders of magnitude lower than this.

The large number of infections estimated in our analysis, based on a small number of positive sera, implies a substantial risk of exposure to infection among the local population of Guangzhou. Visits to live poultry markets are common in Guangzhou, with one survey in May–August 2013 finding that 47 % of adult respondents had visited a live poultry market at least once in the preceding year [12]. A previous serological study in Shenzhen identified serological evidence of high rates of infection (7.2 % in May 2013 and 14.9 % in December 2013) in poultry workers but not in the general population, consistent with repeated occupational exposures [14]. In that study, the absence of confirmed infections or severe illnesses in poultry workers with serological evidence of recent infection is also consistent with most infections being mild. A study in Beijing estimated very low risks of H7N9 in poultry workers in Beijing, and did not identify any infections in 1300 person-years of exposure among the general population from 2013 to 2015 [15].

## Conclusion

In conclusion, by estimating the number of human infections with influenza A(H7N9) virus in Guangzhou between January 2014 and April 2014, we were able to show that most infections are not associated with severe disease, which is consistent with the interpretation of mild H7N9 cases detected through ILI surveillance [3, 10, 16, 17]. However, it is concerning that so many mild infections have occurred, implying that H7N9 has had many opportunities to acquire specific adaptations needed for efficient transmission of H7N9 between humans.

## Additional file

Additional file 1: Technical Appendix (DOCX 51 kb)

## Abbreviations

CrI: Credibility interval
HI: Haemagglutination inhibition
MCMC: Markov Chain Monte Carlo
