# Erratum to: Population seroprevalence of antibody to influenza A(H7N9) virus, Guangzhou, China

**DOI:** 10.1186/s12879-017-2359-z

**Published:** 2017-04-19

**Authors:** Yong Ping Lin, Zi Feng Yang, Ying Liang, Zheng Tu Li, Helen S. Bond, Huiying Chua, Ya Sha Luo, Yuan Chen, Ting Ting Chen, Wen Da Guan, Jimmy Chun Cheong Lai, Yu Lam Siu, Si Hua Pan, J. S. Malik Peiris, Benjamin J. Cowling, Chris Ka Pun Mok

**Affiliations:** 1 Department of Laboratory Medicine, The First Affiliated Hospital of Guangzhou Medical University, Guangdong, China; 2 Research Centre of Translational Medicine, The First Affiliated Hospital of Guangzhou Medical University, Guangdong, China; 3 State Key Laboratory of Respiratory Disease, National Clinical Research Center for Respiratory Disease, First Affiliated Hospital of Guangzhou Medical University, Guangdong, China; 4 WHO Collaborating Centre for Infectious Disease Epidemiology and Control, School of Public Health, Li Ka Shing Faculty of Medicine, The University of Hong Kong, Hong Kong Special Administrative Region, China; 5 Department of Laboratory Medicine, The Second Affiliated Hospital of Guangzhou University of Chinese Medicine, Guangdong, China; 6 Department of Pathology, Li Ka Shing Faculty of Medicine, The University of Hong Kong, Hong Kong Special Administrative Region, China; 7 HKU-Pasteur Research Pole, School of Public Health, Li Ka Shing Faculty of Medicine, The University of Hong Kong, Hong Kong Special Administrative Region, China; 8 Centre of Influenza Research, School of Public Health, Li Ka Shing Faculty of Medicine, The University of Hong Kong, Hong Kong Special Administrative Region, China; 9 School of Public Health, Li Ka Shing Faculty of Medicine, The University of Hong Kong, 21 Sassoon Road, Pokfulam, Hong Kong, China

## Erratum

After the publication of this work [1] it was noticed that there is a typographical error in the author name ‘Chris Ka Pun Mok’ where the space between the Given Name ‘Pun’ and the Family Name ‘Mok’ has been deleted.
