# Supplementary material for: Population seroprevalence of antibody to influenza A(H7N9) virus, Guangzhou, China
Source: BMC Infect Dis. 2016 Nov 4;16:632. doi: 10.1186/s12879-016-1983-3 (PMC5097368; doi:10.1186/s12879-016-1983-3)
Supplement: Additional file 1: — Technical Appendix (DOCX 51 kb) [file 12879_2016_1983_MOESM1_ESM.docx]

**TECHNICAL APPENDIX**

**Additional details of the inferential framework for estimation of the number of human infections with avian influenza A(H7N9) virus in Guangzhou, China, winter 2013-14**

This appendix provides additional technical details of the statistical methods used to infer cumulative incidence of H7N9 infection and severity of infections, based on serologic surveillance done in Guangzhou, China in 2014. We used notations in Table S1 in describing our methodology. We estimated model parameters in a Bayesian framework. This was implemented using the 'STAN' package in R.

Table S1: The list of parameters with known values and parameters to be estimated by the model.

| **Parameters** | **Definition** | **Value** |
| --- | --- | --- |
| Known |  |  |
| $n_{i}$ | Number of serum samples collected in week $i$ | Refer to Table S2 |
| $y_{i}$ | Number of positive serum samples in week $i$ | Refer to Table S2 |
| ${x'}_{i}$ | Expected scaled seroprevalence, based on a lag between the cumulative incidence of infection and seropositivity, and allowing for possible waning in antibody over a longer time frame. | Refer to Table S2 |
| $M$ | Population in Guangzhou, China | 12,800,000 |
| $D$* | Number of death cases | 11 |
| $S$* | Number of severe cases | 16 |
|  |  |  |
| To be estimated |  |  |
| $p_{i}$ | Probability of infection with influenza A(H7N9) in week $i$, defined as having a seroprevalence at a titer $\geq$40 |  |
| $\theta$ | Cumulative incidence of influenza A(H7N9) infection in the population |  |
| $\theta_{0}$ | Baseline seroprevalence of cross-reactive influenza A(H7N9) antibody (i.e. not due to recent H7N9 infections) |  |
| $in$ | Estimated number of infections ($\theta*M)$ |  |
| $is$ | Estimated infection-severity risk (ISR) |  |
| $if$ | Estimated infection-fatality risk (IFR) |  |

Note: * indicates parameters with values based on reference [1].

The data for weeks in which serum samples were collected for the five different age groups is shown below (Table S2). Week $i$ corresponds to the week starting from 8 December 2013.

Table S2: The number of serum samples collected and tested positive by week and age group.

| Week $i$ | ${x'}_{i}$ | Age Group 1 (0-14 yrs) | | Age Group 2  (15-24 yrs) | | Age Group 3  (25-54 yrs) | | Age Group 4  (55-64 yrs) | | Age Group 5  (65+ yrs) | |
| --- | --- | --- | --- | --- | --- | --- | --- | --- | --- | --- | --- |
|  |  | $n_{i}$ | $y_{i}$ | $n_{i}$ | $y_{i}$ | $n_{i}$ | $y_{i}$ | $n_{i}$ | $y_{i}$ | $n_{i}$ | $y_{i}$ |
| 1 | 0.0000 | 16 | 0 | 16 | 0 | 29 | 0 | 17 | 0 | 18 | 0 |
| 3 | 0.0000 | 13 | 0 | 23 | 0 | 24 | 0 | 10 | 0 | 7 | 0 |
| 5 | 0.0000 | 16 | 0 | 20 | 0 | 26 | 0 | 14 | 0 | 10 | 0 |
| 7 | 0.0000 | 30 | 0 | 24 | 0 | 42 | 0 | 39 | 0 | 5 | 0 |
| 10 | 0.1875 | 31 | 0 | 21 | 0 | 125 | 1 | 54 | 0 | 140 | 0 |
| 11 | 0.2500 | 19 | 0 | 38 | 0 | 149 | 0 | 36 | 0 | 52 | 0 |
| 12 | 0.3125 | 18 | 0 | 35 | 0 | 162 | 0 | 61 | 0 | 87 | 0 |
| 13 | 0.6250 | 32 | 0 | 29 | 0 | 208 | 0 | 44 | 0 | 73 | 0 |
| 14 | 0.8750 | 13 | 0 | 24 | 0 | 130 | 0 | 30 | 0 | 35 | 0 |
| 15 | 0.9375 | 3 | 0 | 16 | 0 | 134 | 0 | 37 | 0 | 35 | 0 |
| 17 | 1.0000 | 36 | 0 | 14 | 0 | 70 | 0 | 21 | 0 | 63 | 1 |
| 19 | 1.0000 | 19 | 0 | 11 | 0 | 85 | 0 | 3 | 0 | 82 | 0 |
| 46 | 0.3125 | 24 | 0 | 37 | 0 | 300 | 0 | 41 | 0 | 62 | 0 |
| 48 | 0.1875 | 47 | 0 | 86 | 0 | 781 | 0 | 213 | 0 | 208 | 0 |
| 49 | 0.0625 | 46 | 0 | 100 | 0 | 191 | 0 | 128 | 0 | 162 | 0 |
| 51 | 0.0000 | 27 | 0 | 19 | 0 | 4 | 0 | 19 | 0 | 116 | 0 |
| 52 | 0.0000 | 14 | 0 | 10 | 0 | 0 | 0 | 0 | 0 | 51 | 0 |

Note: ${x'}_{i}$ = expected scaled seroprevalence; $n_{i}$ = number of serum samples collected in week $i$; $y_{i}$ = number of positive serum samples in week $i$.

Based on the cumulative probability of a severe infection taken from Chen et al. [1] that reported 16 severe cases between January 2014 and March 2014, and the assumption of a 2-week lag period between infection and the development of antibody at titers ≥40 , we calculated the expected scaled seroprevalence, ${x'}_{i}$. We fitted the model using the following four alternative assumptions of ${x'}_{i}$, as illustrated in Figure S1.

1. Antibody titers wane exactly 6 months (26 weeks) after infection (primary analysis as reported in the main text).
2. Infection incidence drops back down to baseline incidence 10 weeks after severe infection occurred.
3. Infection incidence stays constant after all severe infections occurred.
4. Excluding serology data in phase 2 as no positive cases or severe infections occurred in this phase.

Figure S1: The values of ${x'}_{i}$ over time, comparing four alternative assumptions of ${x'}_{i}$.

For each age group, we used the following binomial likelihood for the number of positive specimens observed among the total number of specimens tested each week, to estimate the probability of infection, $p_{i}$.

$$L(p_{i}|n_{i},y_{i})\propto{p_{i}}^{y_{i}}{(1-p_{i})}^{n_{i}-y_{i}}$$

In each age group, $p_{i}$ was set to be equal to $\theta_{0}+ \theta*{x'}_{i}$ , where $\theta_{0}$ is the baseline seroprevalence and $\theta$ is the cumulative incidence of influenza A(H7N9), both of which were to be estimated.

Based on Yang et al. [2] study which found 9 positive results (with titer ≥40) among 1129 individuals of all ages, we calculated age-specific priors for $\theta_{0}$ using the weighted age distribution in Table S3, assuming that the age distribution of samples matched the age distribution of the population (an age-specific breakdown was not provided in that publication).

Table S3: Age-specific priors for baseline seroprevalence, $\theta_{0}$ were calculated using weighted age distributions, from the previous study that shows 9 positive results among 1129 individuals of all ages.

| Age Group | Weight | Prior for baseline seroprevalence, $\theta_{0}$ |
| --- | --- | --- |
| 1: 0-14 yrs | 0.171 | Beta(1.5, 193.1) |
| 2: 15-14 yrs | 0.147 | Beta(1.3, 166.0) |
| 3: 25-54 yrs | 0.472 | Beta(4.2, 532.9) |
| 4: 55-64 yrs | 0.113 | Beta(1.0, 127.6) |
| 5: 65+ yrs | 0.096 | Beta(0.9, 108.4) |

We also specified a Jeffrey’s non-informative prior (i.e. a beta(0.5, 0.5) distribution), for $\theta$. The value for $\theta$ was first estimated for each age group by our model and combined using population weights in Table S3 to calculate a single estimate of the overall age-standardized cumulative incidence of H7N9. This value was then multiplied by the population size of Guangzhou (M=12.8 million) to estimate the total number of infections, $in$.

Finally, using $in$ as the denominator, information of observed number of severe (S) and death case (D), we also estimated the infection-severity risk (ISR) (represented as $is$) and infection-fatality risk (IFR) (represented as $if$) using the following respective binomial likelihoods.

$$L(is|in,S)\propto{is}^{S}{(1-is)}^{in-S}$$

$$L(if|in, D)\propto{if}^{D}{(1-if)}^{in-D}$$

We used the R STAN syntax (as provided below) to conduct main analysis, based on the two sera that had both an HI titer of ≥40 and a neutralization titer ≥40. The main analysis was repeated to consider alternative assumptions of ${x'}_{i}$. We also carried out sensitivity analysis to consider an alternative prior beta distribution of $\theta$ and three sera that were tested positive for H7N9 at a HI titer of ≥40 using the model shown in the main text.

Our model fitted using the Markov Chain Monte Carlo (MCMC) methods with a warmup period of 30,000 iterations and a further 30,000 iterations for sampling and 5 chains showed that the MCMC chains were well-mixed and converged by examination of trace plot and the potential scale reduction statistic. Results were as described in the main text.

The R STAN syntax for the main analysis for age group 1 is provided below to permit reproducibility of our results (similar syntax used for age groups 2-5).

**R STAN syntax**:

data <- list(G = 5, N = length(data_table$week), alpha = c(1.5, 1.3, 4.2, 1.0, 0.9),
 beta = c(193.1, 166.0, 532.9, 127.6, 108.4), n = c(data_table$agegp1_n1,

data_table$agegp2_n2, data_table$agegp3_n3, data_table$agegp4_n4,

data_table$agegp5_n5), y = c(data_table$agegp1_y1, data_table$agegp2_y2,

data_table$agegp3_y3, data_table$agegp4_y4, data_table$agegp5_y5),

x1 = data_table$xi, w = c(0.171, 0.147, 0.472, 0.113, 0.096), S = 16, D = 11)

# x2, x3 and x4 can be used for the alternative formulations described above

#x2 <- c(0,0,0,0,0.1875,0.25,0.3125,0.625,0.875,0.9375,1,1,0,0,0,0,0)

#x3 <- c(0,0,0,0,0.1875,0.25,0.3125,0.625,0.875,0.9375,1,1,1,1,1,1,1)

#x4 <- c(0,0,0,0,0.1875,0.25,0.3125,0.625,0.875,0.9375,1,1) #N=12

stan_code<-"

data {

int G; // number of age groups

  int N; // number of weeks where serum samples were taken

  int n[G*N]; // number of weekly serum samples

  int y[G*N]; // number of positive weekly serum samples

  real x1[N]; // expected scaled seroprevalance

real alpha[G]; // alpha for age-specific prior theta_0

real beta[G]; // beta for age-specific prior theta_0

real w[G]; // age-specific population weight

  int S; // number of severe infections

  int D; // number of deaths

}

parameters {

  real <lower=0, upper=1> theta[G]; // cumulative incidence of infections (age-specific)

    real <lower=0, upper=1> theta_0[G]; // baseline cumultive incidence of infections (age-specific)

    simplex[2] est_ifr; // estimated infection-severity risk

    simplex[2] est_isr; // estimated infection-fatality risk

}

transformed parameters {

    matrix<lower=0, upper=1>[G,N] p; // probability of infection

real weighted_theta[G]; // weighted theta for each age group

real stan_theta; // overall age-standardised theta

    real est_inf; // estimated total number of infections

for (i in 1:G)

for (j in 1:N)

p[i,j] <- inv_logit(theta0[i] + theta[i] * x1[j]); // inv_logit transforms p to values in the interval [0,1]

for (i in 1:G)

weighted_theta[i] <- theta[i] * w[i];

stan_theta <- sum(weighted_theta)/(0.171+0.147+0.472+0.113+0.096);

est_inf <- stan_theta * 12.8e6; // total population size for Guangzhou = 12.8e6

}

model {

    real Pr_D;

    real Pr_S;

    theta ~ beta(0.5, 0.5); // prior for theta

    theta_0 ~ beta(alpha, beta); // prior for theta_0

    est_ifr ~ beta(0.5, 0.5); // prior for est_ifr

    est_isr ~ beta(0.5, 0.5); // prior for est_isr

for (i in 1:G)

for (j in 1:N)

y[i*j] ~ binomial(n[i*j], p[i,j]); // binomial likelihood of a positive serum sample

  Pr_S <-  binomial_coefficient_log(est_inf, S)

   +multiply_log(S, est_isr[1])

   +multiply_log((est_inf-S), est_isr[2]); // likelihood for Pr_S

    increment_log_prob((Pr_S));

Pr_D <-  binomial_coefficient_log(est_inf, D)

    +multiply_log(D, est_ifr[1])

    +multiply_log((est_inf-D), est_ifr[2]); // likelihood for Pr_D

    increment_log_prob((Pr_D));

// binomial_coefficient_log and multiple_log function was used instead of the more commonly used binomial function, due to the design of RSTAN package that does not allow N (equivalent to est_inf in our model) to be unknown ahead of time and to be a real number.

}"

fit.estimate <- stan(model_code=stan_code, data=data, iter=6e4, chains=5)

**Appendix References**

1. Chen, Z., et al., Asymptomatic, mild, and severe influenza A(H7N9) virus infection in humans, Guangzhou, China. Emerg Infect Dis, 2014. 20(9): p. 1535-40.

2. Yang, S., et al., Avian-origin influenza A(H7N9) infection in influenza A(H7N9)-affected areas of China: a serological study. J Infect Dis, 2014. 209(2): p. 265-9.
